# High-dose proton beam therapy versus conventional fractionated radiation therapy for newly diagnosed glioblastoma: a propensity score matching analysis

**DOI:** 10.1186/s13014-023-02236-1

**Published:** 2023-02-23

**Authors:** Masahide Matsuda, Masashi Mizumoto, Hidehiro Kohzuki, Narushi Sugii, Hideyuki Sakurai, Eiichi Ishikawa

**Affiliations:** 1 Department of Neurosurgery, Faculty of Medicine, University of Tsukuba, Tsukuba, Ibaraki 305-8575 Japan; 2 Department of Radiation Oncology, Proton Medical Research Center, University of Tsukuba, Tsukuba, Ibaraki Japan

**Keywords:** Proton beam therapy, Glioblastoma, Treatment outcome, Propensity score matching

## Abstract

**Background:**

High-dose proton beam therapy (PBT) uses excellent dose concentricity based on the unique characteristic termed the Bragg peak. PBT is a highly feasible treatment option that improves survival in select patients with newly diagnosed glioblastoma (GBM). However, selection bias remains an issue in prior studies that evaluated the efficacy of PBT. The aim of the present study was to compare the survival outcomes and toxicities of high-dose PBT and conventional radiation therapy (CRT) using propensity score-matched treatment cohorts.

**Methods:**

The analysis included patients with newly diagnosed GBM treated with high-dose PBT of 96.6 Gy (RBE) or CRT of 60 Gy from 2010 to 2020. Propensity score generation and 1:1 matching of patients were performed based on the following covariates: age, sex, tumor location, extent of resection, chemotherapy, immunotherapy, and pre-radiation Karnofsky performance scale score.

**Results:**

From a total of 235 patients, 26 were selected in each group by propensity score matching. The median overall survival (OS) of the PBT group was 28.3 months, while the median OS of the CRT group was 21.2 months. Although acute radiation-related toxicities were equivalent between the PBT and CRT groups, radiation necrosis as a late radiation-related toxicity was observed significantly more frequently in the PBT group.

**Conclusions:**

High-dose PBT provided significant survival benefits for patients with newly diagnosed GBM compared to CRT as shown by propensity score matching analysis. Radiation necrosis remains an issue in high-dose PBT; thus, the establishment of an effective treatment strategy centered on bevacizumab would be essential.

## Introduction

Glioblastoma (GBM) is the most aggressive type of brain tumor and has a poor prognosis; the median survival of patients is 14.6 months despite maximal safe resection followed by chemoradiotherapy using temozolomide [1]. To improve GBM patient survival, many therapeutic strategies have been explored over the past few decades. Radiation dose escalation using new techniques such as particle therapy or intensity-modulated radiation therapy (IMRT) represents a promising strategy for localized treatment of GBM.

We developed dose-escalated radiation therapy with a total dose of 96.6 Gy [relative biological effectiveness (RBE)] using a proton beam [2]. Proton beam therapy (PBT) enables high-dose irradiation of tumors without increasing the dose to the surrounding normal tissue by applying a sharp energy peak called the Bragg peak [3]. We previously reported that high-dose PBT is feasible and improves survival in selected patients with newly diagnosed GBM [2, 4, 5]. However, the influence of selection bias related to the inclusion criteria of prior clinical studies on PBT must be addressed to accurately evaluate the benefits of PBT compared with those of conventional radiation therapy (CRT).

To address confounding factors related to selection bias, we performed propensity score matching analysis. Propensity score matching techniques reduce bias in estimating therapeutic effects when analyzing nonrandomized observational data [6]. The aim of the present study was to compare the survival outcomes and toxicities of high-dose PBT versus CRT using propensity score-matched treatment cohorts.

## Materials and methods

We retrospectively analyzed 235 consecutive patients with newly diagnosed GBM who were treated with radiation therapy of ≥ 60 Gy at the University of Tsukuba Hospital from January 2010 to June 2020. All 235 patients underwent different extents of surgical resection: gross total resection (GTR) for the complete resection of contrast-enhancing tumor, subtotal resection (STR) for tumor resection of 90% or more, partial resection (PR) for tumor resection of 5% to 90%, and biopsy (B) for tumor resection of less than 5% and were diagnosed with GBM by histopathological examination based on the classification system of the World Health Organization.

All patients received one of two different modalities of radiation therapy following surgery. For CRT, a total dose of 60 Gy in 30 fractions was delivered by daily photon radiation (2.0 Gy administered five times per week). For high-dose PBT, a total dose of 96.6 Gy (RBE) in 56 fractions by hyperfractionated concomitant boost was delivered [2]. Proton beams with an energy of approximately 250 MeV were produced using a booster synchrotron at the Proton Medical Research Center. For the clinical application of PBT, we adopted a RBE of 1.1. A total dose of 96.6 Gy (RBE) was prescribed for GTV_96.6 Gy_ defined as the area of contrast-enhanced tumor or surgical cavity on magnetic resonance imaging (MRI) plus a 5 mm margin. CTV_73.5 Gy_ and CTV_50.4 Gy_ were defined as the area of a 15 mm margin around contrast-enhancing tumor or surgical cavity and the area of 20 mm margin around peritumoral edema, respectively. The exposure dose was limited to less than 50 Gy (RBE) for the chiasm and less than 60 Gy (RBE) for the thalamus and brainstem. The application of PBT was dependent on eligibility based on inclusion criteria and patient decision. The main inclusion criteria included unlike fatality of the predicted radiation necrosis and potential resectability of brain necrosis within GTV_96.6 Gy_ [4].

For postoperative chemotherapy, temozolomide (TMZ) was administered in most patients according to the modified Stupp regimen (concomitantly with postoperative radiation therapy and then adjuvant maintenance therapy for 12 to 24 cycles). In several patients, autologous formalin-fixed tumor vaccine (AFTV) was administered as immunotherapy, in addition to postoperative chemoradiotherapy. The detailed procedure of AFTV has been previously described [7].

Molecular analysis of tumor tissue was performed only in a limited number of cases. O^6^-methylguanine-DNA-methyltransferase (MGMT) promoter methylation status was analyzed using methylation-specific polymerase chain reaction, and isocitrate dehydrogenase-1 (IDH1) R132H mutation status was assessed using immunohistochemistry. Adverse events were evaluated according to the Common Terminology Criteria for Adverse Events (CTCAE) v5.0. Radiation necrosis was diagnosed mainly by MRI and ^11^C- methionine-positron emission tomography (MET-PET) imaging. Only cases for whom surgery was performed were diagnosed pathologically. Imaging radiation necrosis was defined as new contrast enhancement without high uptake in MET-PET within the previous irradiation fields.

The study protocol was approved by the Ethics Committee of the University of Tsukuba Hospital (number R01-202).

Statistical analyses were performed using SPSS version 28 (SPSS, Inc.). Propensity score generation and 1:1 matching of patients were performed between the CRT and PBT groups to minimize selection bias in treatment allocation. Matching was based on seven covariates for their potential association with survival outcomes: age, sex, tumor location, extent of resection, chemotherapy, immunotherapy, and pre-radiation Karnofsky performance scale (KPS) score. Overall survival (OS), defined as the time from surgery until death, was calculated using Kaplan–Meier analysis. Progression-free survival (PFS), defined as the time from surgery until progression, was calculated using Kaplan–Meier analysis. Necrosis-free survival, defined as the time from radiation until the detection of radiation necrosis, was calculated using Kaplan–Meier analysis. The log-rank test was used to evaluate differences in survival outcomes between the CRT and PBT groups. Continuous and categorical variables were compared between the two groups using Student’s t-test and Fisher’s exact test, respectively. Data were considered statistically significant at *p* < 0.05.

## Results

The baseline characteristics of the 235 patients are summarized in Table 1. Of the 235 patients, 26 were treated with high-dose PBT and 209 with CRT following tumor resection. In the original cohort, significant differences were detected between the two groups in the extent of resection, pre-radiation KPS scores, and MGMT promoter methylation status. More patients in the PBT group underwent GTR or STR and had pre-radiation KPS scores ≥ 70 than patients in the CRT group. After matching for covariates including age, sex, tumor location, extent of resection, chemotherapy, immunotherapy, and pre-radiation KPS score, the best final match of 26 patients was generated. Molecular information, including MGMT promoter methylation status and IDH1 mutation status, was not included in the propensity score model because this information was only available for a limited number of cases. The baseline characteristics of the propensity-score-matched patients are summarized in Table 1. The distributions of the seven covariates were balanced between the two groups. In addition, no significant differences in molecular information were found between the two matched groups.

**Table 1:** Patients characteristics stratified by cohort (PBT and CRT) before and after propensity matching

	Before propensity score matching (n = 235)			After propensity score matching (n = 52)		
	PBT (n = 26)	CRT (n = 209)	*p* value	PBT (n = 26)	CRT (n = 26)	*p* value
Age (y), mean ± SD	57.6 ± 12.3	62.5 ± 13.1	0.075	57.6 ± 12.3	54.3 ± 14.3	0.37
Sex, female (%)	11 (42.3%)	87 (41.6%)	1.000	11 (42.3%)	7 (26.9%)	0.382
Tumor location (cerebral lobe) (%)	25 (96.2%)	171 (81.8%)	0.090	25 (96.2%)	26 (100%)	1
Extent of resection (GTR, STR) (%)	23 (88.5%)	122 (58.4%)	0.002	23 (88.5%)	19 (73.1%)	0.291
Temozolomide (%)	26 (100%)	205 (98.1%)	1.000	26 (100%)	25 (96.2%)	1
Immunotherapy (%)	5 (19.2%)	22 (10.5%)	0.195	5 (19.2%)	6 (23.1%)	1
KPS (pre RT) ≥ 70 (%)	23 (88.5%)	140 (67.0%)	0.025	23 (88.5%)	22 (84.6%)	1
MGMT methyl (%)	1/21 (4.8%)	47/146 (32.2%)	0.009	1/21 (4.8%)	3/18 (16.7%)	0.318
IDH mut (%)	3/23 (13.0%)	9/181 (5.0%)	0.140	3/23 (13.0%)	1/18 (5.6%)	0.618

*GTR* Gross total resection, *STR* Subtotal resection, *KPS* Karnofsky performance scale, *MGMT* O6-methylguanine-DNA-methyltransferase, *IDH* isocitrate dehydrogenase

**Fig. 1:**
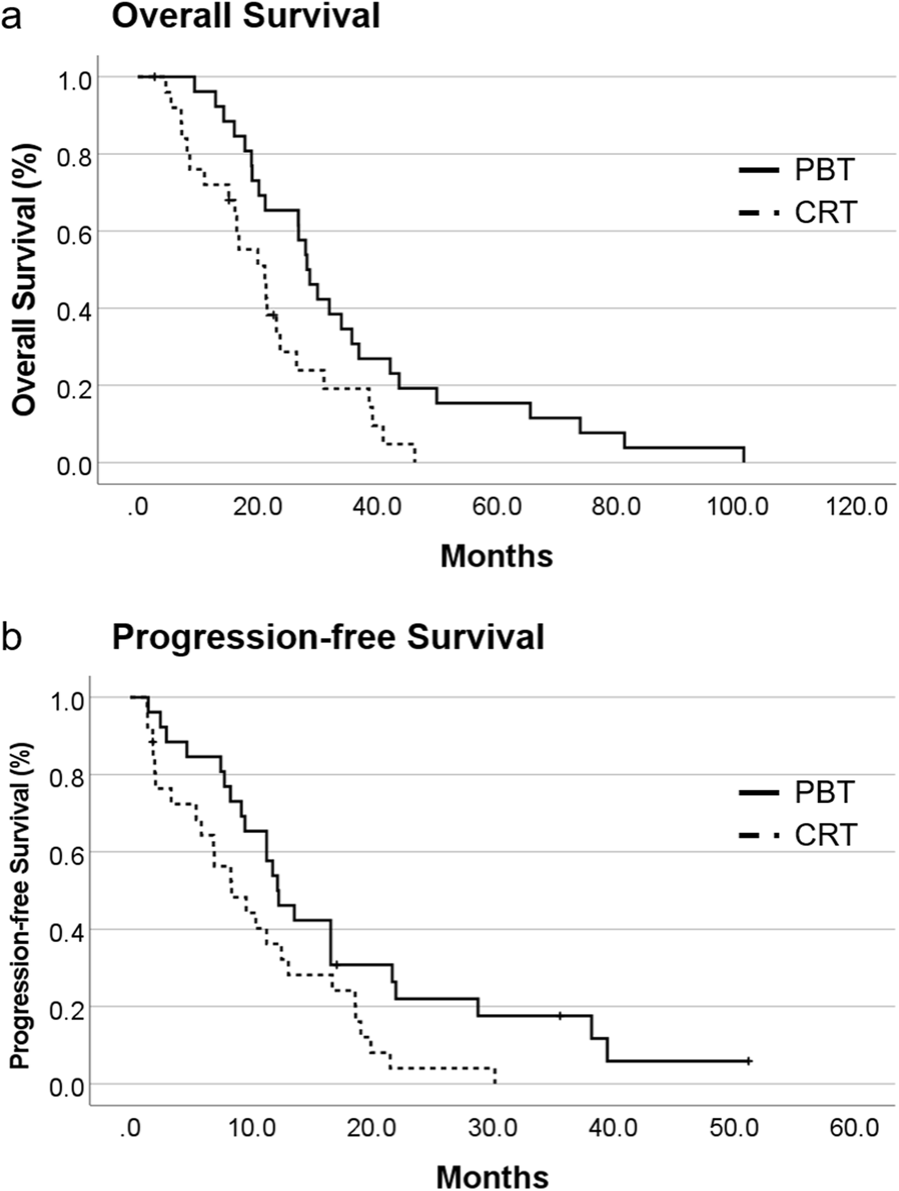
**a** Kaplan–Meier curves of OS according to radiation modality. OS of the PBT group was significantly longer than the OS of the CRT group. **b** Kaplan–Meier curves of PFS according to the radiation modality. PFS of the PBT group was significantly longer than the PFS of the CRT group

**Fig. 2:**
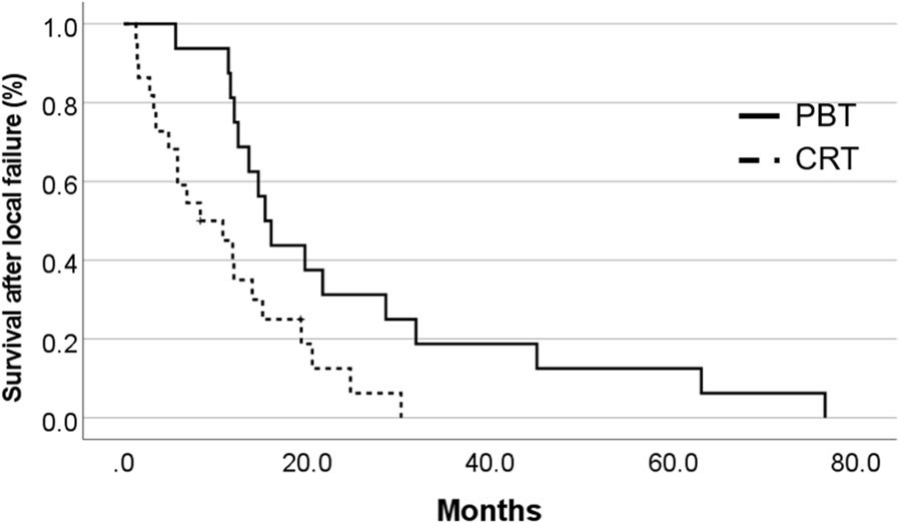
Kaplan–Meier curves of survival after local failure according to radiation modality. Survival time after local failure in the PBT group was significantly longer than that in the CRT group

In the matched cohort, the PBT group had a significantly longer median OS of 28.3 months [95% confidence interval (CI) 24.3–32.2 months] than did the CRT group at median OS of 21.2 months (95% CI 14.2–28.3 months, *p* = 0.013, Fig. 1a). The one- and two-year survival rates in the PBT group were 96.2% and 65.4%, respectively. The one- and two-year survival rates in the CRT group were 72.0% and 28.7%, respectively. Furthermore, the median PFS of the PBT group was 12.2 months (95% CI 9.3–15.1 months), which was significantly longer than the median PFS of 8.4 months (95% CI 4.1–12.7 months) for the CRT group (*p* = 0.029) (Fig. 1b). Regarding failure patterns, 16 local failures, 2 distant failures, and 5 disseminations were observed in the PBT group. In contrast, 22 local failures and 3 disseminations occurred in the CRT group. Regarding local failure, the median survival time after failure was 15.4 months (95% CI 12.7–18.2 months) in the PBT group and 8.4 months (95% CI 1.0–15.7 months) in the CRT group (*p* = 0.011) (Fig. 2).

**Fig. 3:**
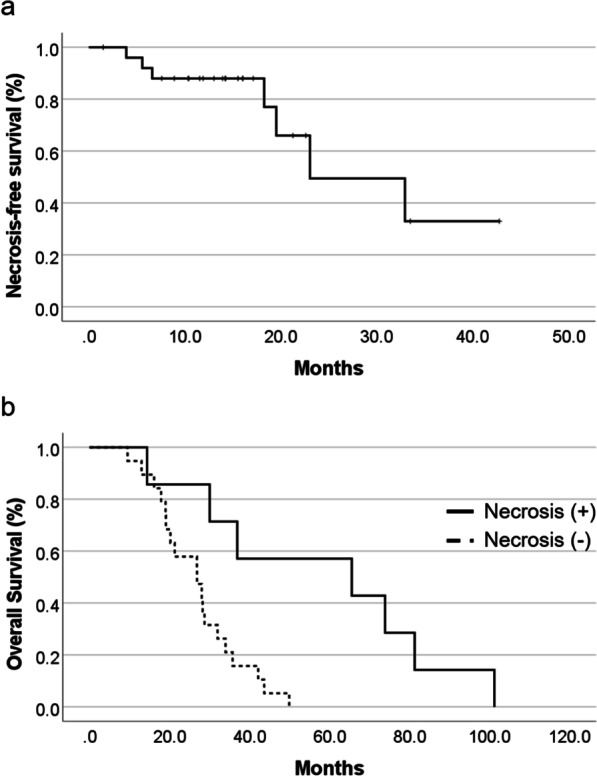
**a** Kaplan–Meier curves of necrosis-free survival. The median time from radiation therapy to development of radiation necrosis was 18.2 months. **b**: Kaplan–Meier curves of OS in the PBT group stratified by coexistence of radiation necrosis. OS of patients with radiation necrosis was significantly longer than the OS of patients without radiation necrosis

For acute radiation-related toxicities, similar frequencies and severities of adverse events were observed in the two groups (Table 2). Temporary alopecia, mostly CTCAE grade 2, was noted in all patients in both groups. Radiation dermatitis was observed in 22 (14 for CTCAE grade 1 and 8 for grade 2) patients in the PBT group and 14 (11 for CTCAE grade 1 and 3 for grade 2) patients in the CRT group. Radiation otitis was observed in 2 patients in the PBT group and 4 patients in the CRT group. Regarding late radiation-related toxicities, radiation necrosis was detected only in the PBT group but not in the CRT group (Table 2). In the PBT group, radiation necrosis was diagnosed in 7 of 26 patients (26.9%). Of these 7 patients (3 for CTCAE grade 1, 2 for grade 2, and 2 for grade 3), 5 patients were symptomatic and treated. Two were treated by resection because tumor recurrence rather than radiation necrosis was suspected preoperatively. In the remaining 3 patients treated medically, conventional therapy including corticosteroids, anticoagulants, or vitamin E was not effective, and all these patients were successfully treated with bevacizumab (Bev). The median time from radiation therapy to the development of radiation necrosis was 23.0 months (95% CI 8.8–37.3 months) (Fig. 3a). In the PBT group, the median OS was 65.6 months (95% CI 0.0–139.0 months) for patients with radiation necrosis and 26.9 months (95% CI 17.3–36.4 months) for patients without radiation necrosis (Fig. 3b). Thus, the complications of radiation necrosis were strongly associated with prolonged survival in patients treated with high-dose PBT (*p* = 0.004).

**Table 2:** Radiation-related toxicity after PBT or CRT

Event	PBT (n = 26)			CRT (n = 26)		
	Grade 1	Grade 2	Grade 3	Grade 1	Grade 2	Grade 3
*Acute toxicities, no.(%)*						
Alopecia	1 (3.8%)	25 (96.2%)	0 (0%)	1 (3.8%)	25 (96.2%)	0 (0%)
Radiation dermatitis	14 (53.8%)	8 (30.8%)	0 (0%)	11 (42.3%)	3 (11.5%)	0 (0%)
Radiation otitis	2 (7.7%)	0 (0%)	0 (0%)	4 (15.4%)	0 (0%)	0 (0%)
*Late toxicities, no.(%)*						
Radiation necrosis	3 (11.5%)	2 (7.7%)	2 (7.7%)	0 (0%)	0 (0%)	0 (0%)

## Discussion

In the present study, we demonstrated that high-dose PBT conferred a statistically significant survival advantage in patients with GBM compared to CRT using propensity-matched cohorts. Although acute radiation-related toxicities were equivalent between the PBT and CRT groups, radiation necrosis as a late radiation-related toxicity was more prevalent in the PBT group.

A proton beam has a unique characteristic termed the Bragg peak, which has a sharp distal dose fall-off in its depth-dose distribution [3]. Owing to its excellent dose concentricity compared with that of conventional fractionated radiation therapy, PBT is advantageous for precise targeting of tumors and reduces doses to nearby critical structures and the surrounding normal tissue [5, 8]. Thus, PBT is widely applied to pediatric tumors to minimize radiation doses to an organ at risk and reduce the risk of future health problems [9]. For GBM, a phase II randomized trial of PBT versus IMRT was performed to assess whether PBT decreased cognitive toxicity compared to IMRT [10]. In that study, the same dose of radiation was delivered to the same tumor target volume in both study arms, and no difference in PFS or OS was observed between the study arms. As a result, PBT was not associated with a delay in time to cognitive failure. The lack of benefit of PBT for cognitive toxicity is probably due to the aggressive nature of GBM which overshadows any potentially improved cognitive outcomes. Conversely, we applied the outstanding target conformity of PBT to deliver higher doses to the tumor while sparing doses to the normal tissues, with the expectation of better local tumor control. We developed a hyperfractionated concomitant boost PBT of 96.6 Gy (RBE) in 56 fractions and showed favorable survival outcomes in patients with GBM [2, 4, 5]. In the current study, high-dose PBT with this protocol was demonstrated to prolong the survival of patients with newly diagnosed GBM compared to CRT. In addition, a randomized trial for newly diagnosed GBM, NRG BN001 (NCT02179086), is ongoing to assess the potential survival benefit of dose-escalated PBT of 75 Gy (RBE) compared to standard-dose photon therapy of 60 Gy.

According to our previous analyses of failure patterns, local tumor control was achieved in areas irradiated with ≥ 90 Gy (RBE), whereas local failure was most frequently observed in areas irradiated with ≤ 60–70 Gy (RBE) [2, 5]. This suggests that 90 Gy (RBE) of irradiation by PBT is the minimum dose required to induce efficient local tumor control of GBM. Regarding the failure patterns in the present study, distant failure or dissemination was more frequently observed in the PBT group than in the CRT group. Furthermore, the survival time after local failure was significantly longer in the PBT group than in the CRT group, suggesting that most local failures in the PBT group were attributed to both recurrent tumors and radiation necrosis, which had relatively lower activity than recurrent tumors alone.

Several other approaches to radiation dose escalation have been investigated for GBM treatment. Nakagawa et al. documented that high-dose conformal radiotherapy of 90 Gy failed to improve the survival of patients with GBM, although local recurrence was significantly decreased compared with that of standard-dose radiotherapy [11]. Chan et al. reported that dose escalation to 90 Gy using the 3D conformal radiation method failed to change the predominant recurrence pattern, and local recurrence remained the most commonly observed pattern [12]. In contrast, Tanaka et al. demonstrated that high-dose conformal radiotherapy of 80–90 Gy in 40–45 fractions provided significant survival benefits over standard 60 Gy radiotherapy without notably increased disability due to complicated radiation necrosis [13]. Collectively, the benefits of high-dose radiation therapy of 90 Gy by standard fractionation have been controversial and remain to be elucidated. Using the hypofractionation method, Iuchi et al. assessed the effect and toxicity of hypofractionated high-dose IMRT of 68 Gy in 8 fractions with concurrent and adjuvant TMZ [14]. Although survival prolongation with a median OS of 20.0 months was achieved, radiation necrosis was observed not only in the high-dose field, but also in the subventricular zone, causing deterioration in the performance status. The potential benefits of high-dose radiation therapy should be carefully balanced against the possible risks associated with dose escalation.

Regarding acute radiation-related toxicities, no significant differences were observed between the PBT and CRT groups. Although radiation dermatitis was slightly more frequent in the PBT group than in the CRT group, the complication rate of dermatitis associated with high-dose irradiation was reduced to this level because of the relatively low entrance doses of PBT. Despite the excellent dose concentricity of proton beams, radiation necrosis as a late radiation-related toxicity remains an issue in high-dose PBT. In our series, radiation necrosis was observed in 7 of 26 patients (26.9%) in the PBT group but not in the CRT group. Although the incidence of radiation necrosis after conventional radiation therapy for glioma is largely unknown, Ruben JD et al. reported the incidence as 4.9% [15]. The reason why no radiation necrosis was observed in the CRT group in our series would be mainly due to relatively small sample size. In the PBT group, although the high rate of radiation necrosis is not negligible, complications of radiation necrosis were significantly associated with better patient survival. Fitzek et al. also described that patients who had radiation necrosis only showed longer survival after PBT compared to those who had a mixture of radiation necrosis and recurrent tumor [16]. Moreover, cases of radiation necrosis diagnosed by imaging may include cases of pseudoprogression that is known as favorable prognostic factor [2]. Of these 7 patients with radiation necrosis, 3 were successfully treated with Bev. Several recent studies have revealed that Bev is a promising alternative treatment for radiation necrosis. Bev can effectively alleviate perilesional edema by restoring the blood–brain barrier and inhibiting angiogenesis via trapping vascular endothelial growth factor [17]. A recent prospective multicenter clinical trial of Bev in patients with symptomatic radiation necrosis demonstrated that Bev immediately alleviated perilesional edema and achieved a high cumulative remission rate [18]. In addition, regarding cases of radiation necrosis following PBT, some previous reports mentioned the effectiveness of Bev [19, 20]. To implement high-dose PBT, it is essential to establish an effective treatment strategy centered on Bev.

The present study has some limitations. The most significant limitation is the lack of an established method for distinguishing tumor recurrence from radiation necrosis. Although we performed MET-PET and perfusion MRI as far as possible for a more accurate diagnosis, the combination of these imagings and pathological diagnostic methods using limited specimens are still insufficient for rigorous differentiation, primarily because various degrees of co-occurrence of recurrent tumors and radiation necrosis exist in the MRI changes with contrast enhancement after high-dose radiation therapy. Also, molecular information was only available for a limited number of cases and not included in the propensity score matching. The other limitations of our study are its retrospective design, relatively small sample size, and remaining bias after propensity score matching. Further studies in large randomized controlled trials are warranted to validate our findings.

## Conclusion

Using propensity score matching analysis, we demonstrated that high-dose PBT provided significant survival benefits for patients with newly diagnosed GBM compared to CRT. Although radiation necrosis caused by high-dose PBT remains an issue, a treatment strategy containing Bev is expected to resolve this issue. Further large-scale prospective randomized studies are warranted to confirm the efficacy of high-dose PBT demonstrated in the current study.

## Data Availability

The datasets used and/or analyzed during the current study are available from the corresponding author on reasonable request.

## Abbreviations

GBM: Glioblastoma
IMRT: Intensity-modulated radiation therapy
RBE: Relative biological effectiveness
PBT: Proton beam therapy
CRT: Conventional radiation therapy
GTR: Gross total resection
STR: Subtotal resection
PR: Partial resection
B: Biopsy
MRI: Magnetic resonance imaging
TMZ: Temozolomide
AFTV: Autologous formalin-fixed tumor vaccine
MGMT: O^6^-methylguanine-DNA-methyltransferase
IDH1: Isocitrate dehydrogenase-1
CTCAE: Common terminology criteria for adverse events
KPS: Karnofsky performance scale
OS: Overall survival
PFS: Progression-free survival
CI: Confidence interval
MET-PET: ^11^C-methionine-positron emission tomography
Bev: Bevacizumab
